# Effect of Text Messaging Parents of School-Aged Children on Outdoor Time to Control Myopia: A Randomized Clinical Trial

**DOI:** 10.1001/jamapediatrics.2022.3542

**Published:** 2022-09-26

**Authors:** Shi-Ming Li, An-Ran Ran, Meng-Tian Kang, Xiaoyuan Yang, Ming-Yang Ren, Shi-Fei Wei, Jia-He Gan, Lei Li, Xi He, He Li, Luo-Ru Liu, Yipeng Wang, Si-Yan Zhan, David A. Atchison, Ian Morgan, Ningli Wang, and the Anyang Childhood Eye Study Group

**Affiliations:** 1 Beijing Institute of Ophthalmology, Beijing Tongren Eye Center, Beijing Tongren Hospital, Capital Medical University, Beijing Key Laboratory of Ophthalmology and Visual Sciences, Beijing, China; 2 Department of Ophthalmology and Visual Sciences, The Chinese University of Hong Kong, Hong Kong, China; 3 Department of Ophthalmology, Henan Provincial People’s Hospital, Zhengzhou, China; 4 School of Mathematical Sciences, Chinese Academy of Sciences, Beijing, China; 5 Key Laboratory of Big Data Mining and Knowledge Management, Chinese Academy of Sciences, Beijing, China; 6 Anyang Eye Hospital, Anyang, China; 7 Department of Epidemiology and Health Statistics, School of Public Health, Peking University, Beijing, China; 8 Centre for Vision and Eye Research, School of Optometry and Vision Science, Queensland University of Technology, Brisbane, Australia; 9 Research School of Biology, Australian National University, Canberra, Australia

## Abstract

**Importance:**

Myopia in school-aged children is a public health issue worldwide; consequently, effective interventions to prevent onset and progression are required.

**Objective:**

To investigate whether SMS text messages to parents increase light exposure and time outdoors in school-aged children and provide effective myopia control.

**Design, Setting, and Participants:**

This randomized clinical trial was conducted in China from May 2017 to May 2018, with participants observed for 3 years. Of 528 965 primary school–aged children from Anyang, 3113 were randomly selected. Of these, 268 grade 2 schoolchildren were selected and randomly assigned to SMS and control groups. Data were analyzed from June to December 2021.

**Interventions:**

Parents of children in the SMS group were sent text messages twice daily for 1 year to take their children outdoors. All children wore portable light meters to record light exposure on 3 randomly selected days (2 weekdays and 1 weekend day) before and after the intervention.

**Main Outcomes and Measures:**

The co–primary outcomes were change in axial length (axial elongation) and change in spherical equivalent refraction (myopic shift) from baseline as measured at the end of the intervention and 3 years later. A secondary outcome was myopia prevalence.

**Results:**

Of 268 grade 2 schoolchildren, 121 (45.1%) were girls, and the mean (SD) age was 8.4 (0.3) years. Compared with the control group, the SMS intervention group demonstrated greater light exposure and higher time outdoors during weekends, and the intervention had significant effect on axial elongation (coefficient, 0.09; 95% CI, 0.02-0.17; *P* = .01). Axial elongation was lower in the SMS group than in the control group during the intervention (0.27 mm [95% CI, 0.24-0.30] vs 0.31 mm [95% CI, 0.29-0.34]; *P* = .03) and at year 2 (0.39 mm [95% CI, 0.35-0.42] vs 0.46 mm [95% CI, 0.42-0.50]; *P* = .009) and year 3 (0.30 mm [95% CI, 0.27-0.33] vs 0.35 mm [95% CI, 0.33-0.37]; *P* = .005) after the intervention. Myopic shift was lower in the SMS group than in the control group at year 2 (−0.69 diopters [D] [95% CI, −0.78 to −0.60] vs −0.82 D [95% CI, −0.91 to −0.73]; *P* = .04) and year 3 (−0.47 D [95% CI, −0.54 to −0.39] vs −0.60 D [95% CI, −0.67 to −0.53]; *P* = .01) after the intervention, as was myopia prevalence (year 2: 38.3% [51 of 133] vs 51.1% [68 of 133]; year 3: 46.6% [62 of 133] vs 65.4% [87 of 133]).

**Conclusions and Relevance:**

In this randomized clinical trial, SMS text messages to parents resulted in lower axial elongation and myopia progression in schoolchildren over 3 years, possibly through increased outdoor time and light exposure, showing promise for reducing myopia prevalence.

**Trial Registration:**

Chinese Clinical Trial Registry Identifier: ChiCTR-IOC-17010525

## Key Points

**Question:**

Do SMS text messages to parents twice daily improve control of myopia in school-aged children?

**Findings:**

In this randomized clinical trial including 268 school-aged children, children in the SMS group had lower myopia progression and reduced myopia prevalence than those in the control group over 2 years.

**Meaning:**

The findings suggest that using text message reminders to parents offers improved control of myopia in school-aged children and should be considered for use with large populations and over large geographic areas.

## Teaser

This randomized clinical trial investigates whether SMS text messages to parents increase light exposure and time outdoors in school-aged children and provide effective myopia control.

## Introduction

In recent decades, myopia has emerged as a major public health issue worldwide.^1^ In Asia,^2,3^ there has been a major increase in myopia prevalence, with lesser increases in European countries^4^ and the US.^5^ A lower age of myopia onset has been observed in recent years compared with the past, leaving more time for myopia progression.^6^ One prediction is that nearly 50% of the world’s population will be myopic by 2050, and nearly 10% will be highly myopic.^7^ In addition, during the COVID-19 pandemic, children aged 6 to 8 years were reported to have significantly increased myopic shifts due to home confinement.^8^ Myopia causes inconvenience and economic burdens in daily life^9^ and may result in severe damage to eyes later in life if it develops into high myopia.^10,11^

Time outdoors has a protective effect in reducing myopia onset and myopic shift,^12,13,14^ confirmed in school-based trials, such as through additional outdoor time during an extended school day^15,16^ or encouraging children to go outside during class recesses.^17^ However, few interventions have been evaluated for increasing time outdoors beyond school hours, such as weekends and holidays. In Singapore, a community-based outdoor intervention program was effective for 6 months^18^ but not to the end of the 9-month trial.^19^ One study found that objectively measured time outdoors for parents and children were correlated,^20^ suggesting that programs promoting the increase of time outdoors in children might be effective using interventions aimed at parents.

On weekdays, Chinese children must do homework after school, often needing to report back to teachers the next day, and it is usually dark when they finish. Consequently, there is little time left to increase outdoor activity on weekdays. On weekends, however, children are looked after by parents who will arrange their activity in detail (including time outside or inside, or time on near work). Therefore, parents can influence children’s time for outdoor activities on weekends.

Phone services, mainly SMS text messages, are now used frequently in public health. SMS messages are effective in helping people quit smoking^21^ and in improving adherence of patients with HIV infection,^22^ asthma,^23^ type 2 diabetes,^24^ and childhood cataract.^25^ WeChat (Tencent Holdings) and other online services are popular in China, with more than 1 billion users, including parents aged 20 to 40 years, who usually have children at risk of myopia.

We hypothesized that text message reminders sent to parents would increase time outdoors and light exposure among school-aged Chinese children and be effective in preventing onset and progression of myopia. We tested this hypothesis with a randomized, investigator-masked intervention trial.

## Methods

### Study Design

The trial was conducted in Anyang Eye Hospital, located in Anyang, Henan province, China, where we have established the Anyang Childhood Eye Study (ACES),^2^ a school-based cohort study to observe myopia in school-aged children. Anyang had 528 965 primary school students. We designed the cohort of the ACES by random selection of 3113 students from 11 primary schools. Grade 2 students from 5 classes were recruited. The trial protocol can be found in Supplement 1.

The trial was approved by the Institutional Review Board of Beijing Tongren Hospital, Capital Medical University, and followed the tenets of the Declaration of Helsinki. Written informed consent was obtained from at least 1 parent after the experimental procedures had been fully described. The trial was registered in the Chinese Clinical Trial Registry (ChiCTR-IOC-17010525) and followed the Consolidated Standards of Reporting Trials (CONSORT) reporting guideline.

### Participants

Children were included according to the following criteria: (1) best-corrected visual acuity of 20/20 OU or better; (2) spherical error ranging from 1.5 diopters (D) to −6.0 D and astigmatism less than 1.5 D in each eye and anisometropia less than 1.0 D; (3) no strabismus, amblyopia, or any other ocular or systematic diseases that may affect refractive development; (4) ability to cooperate with the ocular examinations and questionnaire survey; and (5) not using other interventions to control myopia (for example, acupuncture, massage, drugs, and ear needles) other than school-based eye exercises.^26^

### Randomization, Allocation Concealment, and Masking

Students were allocated by simple randomization (1:1) to the SMS group or to the control group. The randomization sequence was generated using SAS version 9.3 (SAS Institute). The intervention assignment was handed over to an independent staff member, who managed the parents’ cell phone information and contacted the local telecom operator to send text messages regularly to parents in the SMS group. This staff member did not participate in any other process of the trial. The students and study staff, except for those in charge of randomization and sending SMS, were masked to group allocation and measurements.

### Outcomes

The co–primary outcomes were changes in axial length (defined as axial elongation) and cycloplegic spherical equivalent (SE) refraction (defined as myopic shift) from baseline. A secondary outcome was myopia prevalence. An ocular biometry system (Lenstar LS900; Haag-Streit) was used to measure axial length. Five repeated measurements were taken and averaged. Each student was administered a drop of 1% cyclopentolate (Alcon), followed by another drop 5 minutes later. Autorefraction with an HRK-7000A (Huvitz) was performed 30 minutes after the last drop, and 3 measurements were averaged. Refraction was defined as SE (sphere power + cylinder power/2, measured in D), and myopia was defined as SE less than –0.5 D.^27^ Light exposures were measured by a light meter, and time outdoors was defined as illuminance of 1000 lux or greater.^28,29,30^ In normal use, measurements using light meter must fluctuate, with higher values during class recess and after school, for example, and lower values during class and at home. If the illuminance value was fixed, it means that it was not worn, and these measurements were not included for analysis. Axial length and cycloplegic autorefraction were measured before and at the end of the intervention as well as at 2 and 3 years after stopping the intervention. Light exposure was measured before and at the end of intervention.

We derived the total outdoor time for every student from the light meter. The number of 10-second intervals with illuminance of 1000 lux or greater was counted to calculate the total outdoor time in hours (day T = number/360). The illuminance was recorded 8640 times from 7 am to 7 am so that the illuminance per day was the mean of all 10-second counts over 24 hours (day L = sums/8640). The calculation formulas were as follows: weekday T = (day 1 T + day 2 T)/2; weekend T = day 3 T; weekly T = (weekday T × 5) + (weekend T × 2); weekday L = (day 1 L + day 2 L)/2; weekend L = day 3 L; weekly L = (weekday L × 5) + (weekend L × 2).

### Intervention and Procedures

**Figure 1.**
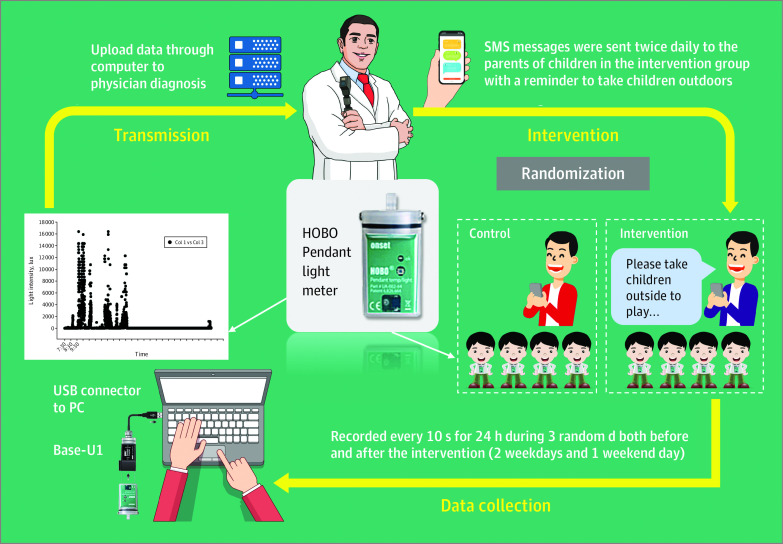
Flow Diagram of Trial Design Including Randomization, Intervention, Data Collection, and Transmission

Figure 1 shows the procedures. During parents’ meetings, all students, parents, and teachers were informed how to wear the light meter and were given eye care knowledge, including the protective effect of increased time outdoors on myopia onset. Some other treatments for controlling myopia were mentioned, such as atropine and orthokeratology, but this would not have affected myopia treatment, as none of these were adopted in children in our study. The children did not wear sunglasses when they were outside, as was determined through the questionnaire survey.

Parents of children in the intervention group received an SMS twice daily on all days of the week, reminding them to take children outdoors. One reminder was sent at noon when children went home for lunch and rested for 2 hours. Another reminder was sent to parents after school time when children had some time available to play outside. For consistency, SMS messages were also sent at these times on weekends. The message was the same every time for parents. Parents in the control group did not receive SMS messages. The study staff kept in close contact with teachers at school. If there was any change for the students, such as parents changing mobile phone numbers, the study staff were informed. If messages were not received, there was feedback from the local communication operator.

Within 2 weeks before the intervention, all children wore a light meter on 3 randomly selected days (2 weekdays and 1 weekend day). This was also done within 2 weeks after the intervention. Under the guidance of parents and teachers, each child wore a waterproof portable light meter (HOBO Pendant UA-002-64; Onset), which was fixed on the clothes, with the light sensor facing outward. The illuminance in lux was recorded every 10 seconds, as has been done in previous studies.^17,30^ Recording time was from 7 am to 7 am on the following day. To help achieve good compliance, we provided free annual ocular examinations and counseling for all children. Consultation was conducted in accordance with the ethical right of the subject to benefit. We answered any questions parents had about the condition of their child’s eyes, but we did not give them any information about the study design.

### Statistical Analysis

Data analysis was performed using the R version 4.0.5 (The R Foundation) on right eyes only. In previous studies, a mean (SD) difference in axial elongation of 0.04 (0.08) mm per year was reported between the children with higher vs lower tertile of time outdoors.^13,15,17,28^ This trial was designed to have a power of 90% and α = .05 to detect the difference in treatment effect. Assuming an estimated loss to follow-up of 20%, at least 105 children were required per group. With a mean (SD) of 0.12 (0.34) D per year for SE refraction,^15,17^ the power for this co–primary outcome was 82.1%.

Mixed-effects models (multilevel models) applicable to longitudinal data were performed, and factors such as time (grade), SMS, and axial length/SE refraction at baseline were incorporated into the model as independent variables to control for random effects about time and to accommodate adjustment for baseline levels (eMethods 1 to 3 in Supplement 2). Change in myopia prevalence between 2 groups was analyzed with χ^2^ tests. The correlation of axial elongation with time outdoors and with time outdoors × light exposure was analyzed using linear regression. The criterion for significance was set at a 2-sided α = .05, and statistical analyses were carried out using R software. A *P* value of .025 (.05/2) was used to control the false-positive rate with co–primary outcomes. Data were analyzed according to the intention-to-treat principle.

## Results

**Figure 2.**
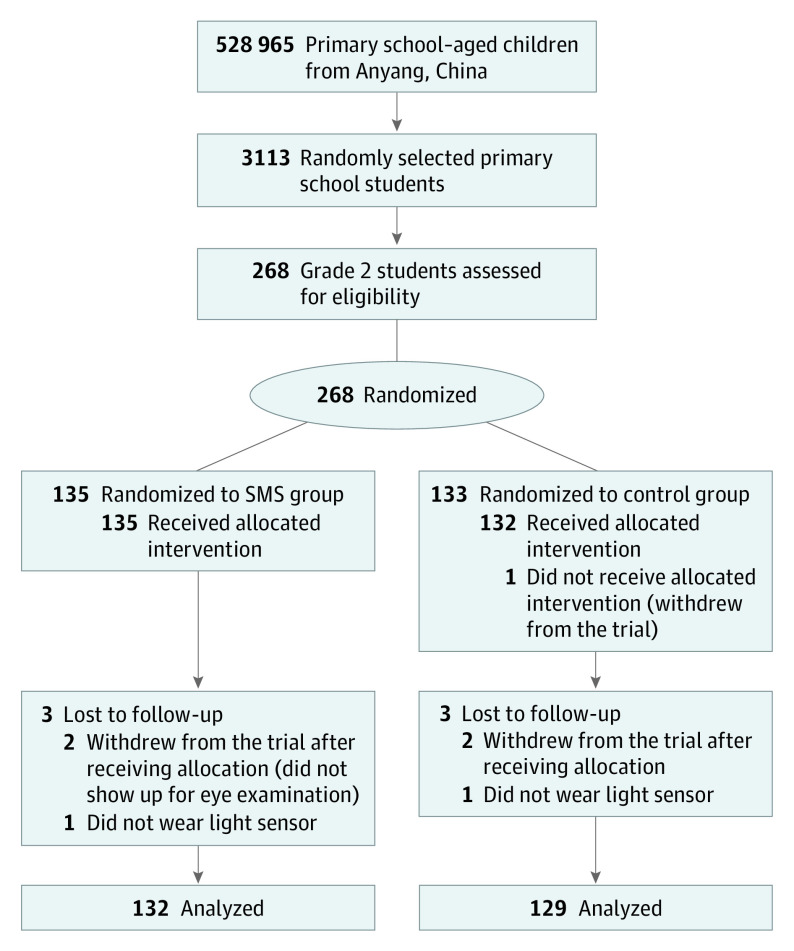
Flow Diagram of Participants According to CONSORT Statement

Figure 2 shows the flow diagram of participants according to the CONSORT statement.^31^ Of 528 965 primary school students, 3113 were selected randomly. Of 268 grade 2 schoolchildren, 121 (45.1%) were girls, and the mean (SD) age was 8.4 (0.3) years. Participants were assigned randomly into the SMS intervention group (n = 135) and the control group (n = 133) between May 2017 and May 2018. Three in the SMS group and 4 in the control group were lost to follow-up. At the end of the trial, 132 in the SMS group and 129 in the control group were available for analysis. The study participants are described in Table 1. The ages in the groups were similar, as were the axial lengths, SE refractions, and other characteristics.

**Table 1.** Baseline Characteristics of Children in This Study

Characteristic	No. (%)	
	SMS group (n = 135)	Control group (n = 133)
Age, mean (SD), y	8.38 (0.34)	8.35 (0.30)
Gender		
Boys	71 (52.6)	76 (57.1)
Girls	64 (47.4)	57 (42.9)
SER, mean (SD), D	0.66 (1.05)	0.37 (1.34)
AL, mean (SD), mm	23.06 (0.69)	23.16 (0.84)
Myopia		
Yes	19 (14.3)	23 (17.3)
No	114 (85.7)	110 (82.7)
Parents with myopia, No.		
0	73 (56.2)	68 (52.7)
1	48 (36.9)	47 (36.4)
2	9 (6.9)	14 (10.9)
Light exposure, mean (SD), lux		
Weekend	57 (27)	57 (26)
Weekday	101 (41)	106 (45)
Weekly	603 (234)	626 (248)
Time outdoors, mean (SD), h		
Weekend	0.45 (0.28)	0.43 (0.24)
Weekdays	0.55 (0.24)	0.58 (0.23)
Weekly	3.54 (1.52)	3.65 (1.39)
Height, mean (SD), m	129.89 (5.42)	128.70 (4.61)
Weight, mean SD), kg	28.7 (5.7)	28.0 (5.5)
Wearing corrective lenses	6 (4.4)	6 (4.5)
DIR, mean (SD), D	−0.58 (1.16)	−0.30 (0.76)

Abbreviations: AL, axial length; D, diopters; DIR, difference in refraction between spherical equivalent in prescriptions and spectacles; SER, spherical equivalent refraction.

Table 2 shows the mixed-effect model for the longitudinal correlation between SMS intervention and primary outcomes as well as adjustment for baseline outcomes, light exposure, and time outdoors. Baseline outcomes and time had the greatest estimated effects on change in axial length and SE refraction. After adjustment for time and baseline outcomes, SMS intervention had a significant effect on change in axial length (coefficient, 0.09; 95% CI, 0.02-0.17; *P* = .01) but not change in SE refraction.

**Table 2.** Parameter Estimates for Mixed-Effect Model for the Outcomes Axial Length and SE Refraction

Measure	Axial length		SE refraction	
	β (95% CI)	*P* value	β (95% CI)	*P* value
Intercept	−1.08 (−2.11 to −0.05)	.04	−0.77 (−2.65 to 1.11)	.42
Time (in y)	0.35 (0.32 to 0.37)	<.001	−0.65 (−0.96 to −0.33)	<.001
SMS	0.09 (0.02 to 0.17)	.01	0.76 (−0.48 to 2.00)	.23
Baseline outcomes^a^	1.04 (0.99 to 1.09)	<.001	1.18 (0.66 to 1.70)	<.001
Light exposure (in lux)	0.0004 (−0.001 to 0.001)	.60	−0.01 (−0.04 to 0.01)	.39
Time outdoors (in h)	−0.02 (−0.29 to 0.25)	.88	3.69 (−1.40 to 8.79)	.16

Abbreviation: SE, spherical equivalent.

^a^ Baseline axial length (mm) or SE refraction (diopters).

Following the 1-year intervention, axial elongation was significantly lower in the SMS group than in the control group (0.27 mm [95% CI, 0.24-0.30] vs 0.31 mm [95% CI, 0.29-0.34]; *P* = .03) (eFigure 1A in Supplement 2). After cessation of the intervention, it remained significantly lower at year 2 (0.39 mm [95% CI, 0.35-0.42] vs 0.46 mm [95% CI, 0.42-0.50]; *P* = .009) and year 3 (0.30 mm [95% CI, 0.27-0.33] vs 0.35 mm [95% CI, 0.33-0.37]; *P* = .005), but not year 4. Among all children, axial elongation was correlated negatively with time outdoors at weekends at year 2 (*r* = −0.12; 95% CI, −0.24 to −0.002) and year 3 (*r* = −0.17; 95% CI, −0.29 to −0.05) as well as with time outdoors × light exposure (year 2: *r* = −0.12; 95% CI, −0.24 to −0.002; year 3: *r* = −0.15; 95% CI, −0.27 to −0.03). As shown in eTable 1 in Supplement 2, children in the SMS group showed greater light exposure and time outdoors than children in the control group at weekends during the intervention.

During the 1-year intervention, myopic shift was lower in the SMS group than in the control group (0.42 D [95% CI, 0.34-0.50] per year vs 0.51 D [95% CI, 0.43-0.59] per year) (eFigure 1B in Supplement 2). After cessation of the intervention, myopic shift was significantly lower at year 2 (0.69 D [95% CI, 0.60-0.78] per year vs 0.82 D [95% CI, 0.73-0.91] per year; *P* = .04) and year 3 (0.47 D [95% CI, 0.39-0.54] per year vs 0.60 D [95% CI, 0.53-0.67] per year; *P* = .01), but not at year 4. Those without myopia had lower axial elongation and myopic shift than those with premyopia and those with myopia over the course of the study (eTable 2 and eFigure 2 in Supplement 2).

Table 3 shows that myopia prevalence was lower in the SMS group than in the control group at year 2 by 12.8% (95% CI, 8.3-24.7; 38.3% [51 of 133] vs 51.1% [68 of 133]), at year 3 by 18.8% (95% CI, 6.9-30.7; 46.6% [62 of 133] vs 65.4% [87 of 133]), and at year 4 by 11.3% (63.9% [85 of 133] vs 75.2% [100 of 133]).

**Table 3.** Prevalence of Myopia in Groups at Different Times

Period	No. (%)		Difference in prevalence, mean (95% CI)	RR (95% CI)
	SMS group (n = 135)	Control group (n = 133)		
Baseline	19 (14.3)	23 (17.3)	3.0 (−5.8 to 11.8)	0.83 (0.43-1.60)
Year 1 (trial period)	33 (24.8)	38 (28.6)	3.8 (−6.9 to 14.4)	0.87 (0.53-1.43)
Year 2	51 (38.3)	68 (51.1)	12.8 (8.3-24.7)	0.75 (0.56-0.99)
Year 3	62 (46.6)	87 (65.4)	18.8 (6.9-30.7)	0.71 (0.57-0.89)
Year 4	85 (63.9)	100 (75.2)	11.3 (0.2-22.3)	0.85 (0.72-1.01)

Abbreviation: RR, relative risk.

## Discussion

In this 1-year randomized clinical trial, children in the SMS group showed less axial elongation, smaller myopic shift in refraction, and lower myopia prevalence than children in the control group. The significant effect of SMS intervention remained at follow-up of years 2 and 3. These findings support the hypothesis that text messages to parents are effective in preventing onset and progression of myopia in children. Using 15 years as an end age and assuming linear rates of change, the estimated reductions of SMS intervention were 0.5 mm for axial length, 1.2 D for myopic shift and 11.7% for myopia prevalence. The decrease in myopia prevalence seems adequate to achieve China’s current short-term national goals for myopia prevention and control. Delaying the onset of myopia is likely to also lower the prevalence of high myopia.

Our findings are consistent with findings from Read et al^28^ that greater daily light exposure (1020 lux or greater) was associated with lower axial elongation over 18 months. With a cutoff value of illuminance 1000 lux or more, Wu et al^17^ found that relatively lower outdoor illuminance with longer time outdoors during the class recess reduced the myopia change in schoolchildren relative to a control group over 1 year, and the axial elongation (0.28 mm per year vs 0.33 mm per year) and myopic shift (0.35 D per year vs 0.47 D per year) were similar to those of our study (axial elongation: 0.27 mm per year vs 0.31 mm per year; myopic shift: 0.42 D per year vs 0.51 D per year). This evidence supports a role for light exposure in the association between time outdoors and myopia development.

It is worth exploring how and when we should increase children’s outdoor time to achieve maximum efficiency in myopia control. A mandatory increase in outdoor time in schools may tempt parents to add more study hours for children after school or on weekends. In our trial, children had more time outdoors on school days than on weekends, probably a consequence of negative feedback from parents. Therefore, it may be necessary to control the activity of private tutorial schools, at least for the early primary school years, which was the double reduction implemented by the Chinese government in 2021. The profound differences in lifestyle between China and Western countries, where children spend more time outdoors on weekends and holidays,^14^ indicates a need for new outdoor activity programs, such as soccer and basketball, for children on weekends and during school holidays.

The SMS intervention in this trial was convenient, economic, and effective, as demonstrated by previous studies on other diseases.^22,23,25^ Consistent with a Singapore trial,^19^ the SMS group in the present trial showed greater light exposure and time outdoors on weekends but not on weekdays, probably due to less time at weekdays available for parents to engage children in outdoor activities. Besides, children were constrained by the same schedule at school, so that the small variations in children’s activities might result in the insignificance on weekdays.

At least 2 factors may contribute to the effectiveness of SMS. First, evidence from previous studies^12,14,15,17^ that getting outdoors helps control myopia in children has been publicized widely in recent years in China, and thus parents are more likely to be persuaded to take children outside. Second, SMS daily could increase compliance, as reported in the treatment of pediatric cataract^25^ and asthma^23^ and in quitting smoking.^21^ Cell phone use is a highly accepted method of communication in China. During the trial, we did not get feedback from parents that SMS messages were annoying. It should be noted that primary and middle school students in Anyang have 2 hours to go home for lunch, and their parents are generally at home at noon. This may not be possible in larger cities. Therefore, school-based interventions within the school program, perhaps during lunch breaks and class recesses,^17^ may be an opportunity to get quality time outdoors on school days.

The prolonged effect of SMS intervention may be because it helps to develop certain habits. Despite having stopped SMS messages for 12 months, axial elongation and myopic shift remained significantly lower in SMS group than in the control group at years 2 and 3. At year 4, all children were in grade 6 and faced the entrance test to junior high school, which may have caused the children and parents to put considerable time into study so that the effect was lost. Further study is warranted to identify the prolonged effect of SMS intervention in myopia control. The equalization of educational resources, such as policy of abolishing key schools (which usually have better teacher and more resources than other schools), may alleviate parents’ anxiety about the later competition for university places and thus help to control myopia.

Trial strengths include cycloplegic autorefraction, simple randomization design, high completion (97.4%), and blind allocation concealment for study staff who measured children and conducted the statistical analyses. The differences in baseline SE and percentage of 2 myopic parents between the groups were not statistically significant. We evaluated the changes, not absolute values, to eliminate potential bias. Additionally, the intervention was delivered via an automated SMS system, providing a highly scalable approach to delivery of theory-based and evidence-based myopia control.

### Limitations

This study has several limitations. First, light exposure and time outdoors might be unrepresentative. To promote good compliance, light exposure was recorded for 3 days only, not the whole year during the intervention, which might lead to random variations originating from school curriculum, family activities, and weather. For example, the weather or the season in Anyang might have some influence on the hours for which illuminance was in excess of the 1000 lux set as the cutoff illuminance of indoors vs outdoors.^19,29,30^ The light meter itself might motivate the children to go outside more in both groups as an incentive. These factors together might explain the inconsistent trends in weekend and weekday time outdoors among children in our trial. Second, the generalizability of the study is limited because of a small sample size and the selection of participants from only grade 2 students. The small sample size may also compromise the statistical power of the co–primary outcomes and may limit our capacity to detect a significant difference on myopic shift between the groups. Masking of parents might not have been completely effective because they could have talked to each other, which could shift the behavior of control group toward intervention group, making it harder to get significant effects.

## Conclusions

This randomized clinical trial showed that text messages twice daily for 1 year prompted parents to encourage grade 2 children to go outside, reducing the decline in outdoor time on weekends from grade 2 to grade 3 and reducing development of myopia in grades 4 and 5. Over the 3 years of our study, increased time outdoors occurred on weekends. This suggests that school-based increases in time outdoors within the school program, perhaps during lunch breaks, may be necessary to increase time outdoors on school days. Text messaging intervention to support myopia prevention programs has the potential to increase efficacy of population-based interventions to prevent and control myopia in children.

## Notes

Supplement 1. Trial Protocol.

Supplement 2. **eMethods 1.** Mixed-Effects Models **eMethods 2.** Structural Equation Model **eMethods 3.** R Script for Performing the Statistical Analysis **eTable 1.** Light Exposure and Time Outdoors Between the 2 Groups Following the Intervention **eTable 2.** Axial Elongation and Myopic Shift Among Subgroups of Nonmyopes, Premyopes, and Myopes During the 1-Year Intervention **eFigure 1.** Axial Elongation (A) and Myopic Shift (B) Following the 1-Year Intervention and for the Next 3 Years **eFigure 2.** Axial Elongation (A) and Myopic Shift (B) Among Subgroups of Nonmyopes, Premyopes, and Myopes Following the 1-Year Intervention and at the Next 3 Years

Supplement 3. The Anyang Childhood Eye Study Group

Supplement 4. Data Sharing Statement
