# Supplementary material for: Effect of Text Messaging Parents of School-Aged Children on Outdoor Time to Control Myopia: A Randomized Clinical Trial
Source: JAMA Pediatr. 2022 Sep 26;176(11):1077–83. doi: 10.1001/jamapediatrics.2022.3542 (PMC9513710; doi:10.1001/jamapediatrics.2022.3542)
Supplement: Supplement 1. — Trial Protocol. [file jamapediatr-e223542-s001.pdf]

PROJECT TITLE

Short Message Service as An Intervention to Increase Time Outdoors in School-aged Children: A Randomized Controlled Trial

Principal Investigator: Ningli Wang, MD, PhD
Professor, Clinician Scientist, Beijing Tongren Eye Center, Capital Medical University, Beijing, China
No 1, DongJiaoMinXiang, DongCheng District, Beijing, 100730

Co-Investigators: Shi-Ming Li, MD, PhD
Associate professor, Project manager, Beijing Tongren Eye Center, Capital Medical University, Beijing, China
No 1, DongJiaoMinXiang, DongCheng District, Beijing, 100730

Si-Yan Zhan, PhD
Professor, Department of Epidemiology and Health Statistics, Peking University School of Public Health, Beijing, China.
No 38, Xueyuan Road, HaiDian District, Beijing, China, 100091

Luoru Liu, MD
President of the hospital, Anyang Eye Hospital, Henan Province, China
East part, WenMingDaDao, Anyang city, Henan Province, China, 455000

He Li, MD
Vice president of the hospital, Anyang Eye Hospital, Henan Province, China
East part, WenMingDaDao, Anyang city, Henan Province, China, 455000

CONTENTS

1. PROTOCOL TEAM ROSTER
2. SITES PARTICIPATING IN THE STUDY
3. BACKGROUND
4. HYPOTHESIS
5. STUDY OBJECTIVES
6. DESIGN AND METHODOLOGY
6.1 Study design
6.2 Study site
6.3 Study populations
6.4 Randomization strategy and sequence generation
6.5 Allocation concealment
6.6 Blinding
6.7 Arms and interventions
6.8 Study outcomes
6.9 Sample size
7. SELECTION AND ENROLLMENT OF SUBJECTS
9.1 Inclusion criteria
9.2 Exclusion criteria
9.3 Study enrollment procedures
8. DATA MANAGEMENT
8.1 Data Collection
8.2 Data Storage and Security
8.3 Statistical Considerations
9. HUMAN SUBJECTS
9.1 Institutional Review Board Review and Informed Consent

9.2 Subject Confidentiality

9.3 Study Discontinuation

10. TIME FRAME

11. REFERENCES

PROTOCOL TEAM ROSTER

Principal Investigator: Ningli Wang, MD, PhD
Professor, Clinician Scientist, Beijing Tongren Eye Center, Capital Medical University, Beijing, China
No 1, DongJiaoMinXiang, DongCheng District, Beijing, 100730

Co-Investigators: Shi-Ming Li, MD, PhD
Associate professor, Project manager, Beijing Tongren Eye Center, Capital Medical University, Beijing, China
No 1, DongJiaoMinXiang, DongCheng District, Beijing, 100730

Si-Yan Zhan, PhD
Professor, Department of Epidemiology and Health Statistics, Peking University School of Public Health, Beijing, China.
No 38, Xueyuan Road, HaiDian District, Beijing, China, 100091

Meng-Tian Kang, MD, PhD
Ophthalmologist, Study co-ordinator, Beijing Tongren Eye Center, Capital Medical University, Beijing, China
No 1, DongJiaoMinXiang, DongCheng District, Beijing, 100730

Xiaoyuan Yang, MD, Ophthalmologist, Department of Ophthalmology, Henan Provincial People’s Hospital, China
No 7, Weiwu Road, Zhengzhou, Henan Province, China, 450003

Luoru Liu, MD
President of the hospital, Anyang Eye Hospital, Henan Province, China
East part, WenMingDaDao, Anyang city, Henan Province, China, 455000

He Li, MD
Vice president of the hospital, Anyang Eye Hospital, Henan Province, China
East part, WenMingDaDao, Anyang city, Henan Province, China, 455000

David A. Atchison, PhD
Professor, School of Optometry & Vision Science and Institute of Health & Biomedical Innovation, Queensland University of Technology, Kelvin Grove Q Australia

Ian Morgan, PhD
Professor, Research School of Biology, Australian National University, Canberra, Australia

Data Management: Shi-Fei Wei, MD
Data management, Beijing Tongren Eye Center, Capital Medical University, Beijing, China
No 1, DongJiaoMinXiang, DongCheng District, Beijing, 100730

An-Ran Ran, MD
Data management, Beijing Tongren Eye Center, Capital Medical University, Beijing, China
No 1, DongJiaoMinXiang, DongCheng District, Beijing, 100730

Statistician: Ming-Yang Ren
School of Mathematical Sciences, University of Chinese Academy of Sciences, Beijing, China
No 19, Yuquan Road, ShiJingShan District, Beijing, China, 100049

Study Nurse: Lili Xu
Anyang Eye Hospital, Henan Province, China, East part, WenMingDaDao, Anyang city, Henan Province, China, 455000

1. SITES PARTICIPATING IN THE STUDY

Beijing Tongren Eye Center, Beijing Tongren Hospital, Capital Medical University

Capital Medical University (CMU) was founded in 1960. It ranks among the top academic medical institutions in China and is one of the key municipal universities in Beijing. Professor Wu Jieping, the founding president of the university, was a world-famous urologist as well as a member of the Chinese Academy of Sciences and the Chinese Academy of Engineering. CMU consists of 10 Schools, 14 affiliated hospitals and 1 teaching institution. The university has over 9,000 enrolled students. CMU provides a wide range of educational programs in English language for Doctorates, Masters, Bachelors and certificates.

CMU is a well-known academic institution for its strong capacity in scientific research. It hosts many key national and municipal disciplines, laboratories and exchange stations for post-doctoral research. High-caliber national and municipal research and training centers and institutes are founded here, covering a wide range of fields including General Practitioner Training, Clinical Medicine, Basic Medicine, Neurosciences, Ophthalmology, Geriatrics, Urology, Cardiology, Pain Medicine, Toxicology, Biomedical Engineering, Traditional Chinese Medicine, Reproduction, Health Policy, etc.

Beijing Tongren Hospital is a famous institution which specializes in ophthalmology and otolaryngology. Beijing Tongren Hospital was founded in 1886 by an American church, Methodist Christ church, which was headquartered in New York at that time. Since its establishment, the hospital has specialized in ophthalmology and the treatment of ear, nose and throat diseases, becoming a recognized authority in these fields.

Beijing Tongren Eye Center is the national key disciplines, doctor's degree authorization centers, national engineering research center for diagnosis and treatment of eye, with its powerful ability of medical and scientific research. It is one of the World Health Organization's blindness collaboration centers in China, and the

supporting unit of the national guide group for preventing blindness of the Ministry of Health. It has set up complete disciplines and has established clinical research and epidemiological research bases in Beijing, Anyang, Handan, Fushun, Shenzhen and Guangdong. It has the experience and data base of organizing and implementing large sample population survey.

Department of Epidemiology and Biostatistics, School of Public Health, Peking University

The department of epidemiology and biostatistics was approved by the ministry of education as a key discipline of universities and colleges in 2002. In the following year, the key laboratory of the Ministry of Education was established as well. The current chair of the department of epidemiology and biostatistics is professor Siyan Zhan.

The main research fields include: Chronic Disease Epidemiology, Infectious Disease Epidemiology, Genetic Epidemiology, Nutritional Epidemiology, Reproductive/Perinatal Epidemiology, Molecular Epidemiology, Systemic Epidemiology, Pharmacological Epidemiology, Clinical Epidemiology and Evidence-based Medicine, Methods in Biostatistics, Drug Abuse Prevention and Control.

From 2010 to 2019, the group has undertaken more than 200 projects which were supported by national natural science foundation of China, Chinese National Programs for Science and Technology Development, Beijing natural science foundation and international cooperation, with a total scientific research fund of over 90 million yuan. In the past three years, 355 research papers have been published including 169 SCI papers of which 21 papers has an impact factor more than 10.

Anyang Eye Hospital

Anyang eye hospital was founded in 1943, after more than 70 years of hard work, has developed into a collection of medical treatment, teaching, scientific research, first aid, prevention, rehabilitation as one of the eye hospitals. In 2009, in order to meet the

development needs of modern ophthalmology, approved by Henan provincial health department, Anyang optometry center was formally established as the second name of our hospital, signifying that the modern ophthalmology system combining ophthalmology and optometry in our city has entered a higher platform.

2. BACKGROUND

Myopia is a public health issue worldwide, especially in Asian countries such as China, Singapore and Japan.[1] Even in the United States, the prevalence of myopia in adults has risen from 25% to 42% from 1972 to 2002, with high myopia also increasing substantially.[2] Myopic retinopathy, which occurs in about 40% of highly myopic eyes,[3] has been reported to be the second leading cause of blindness and low vision in Chinese, ranging from 7.7%-32.7%[4-6]. Meanwhile, myopic retinopathy is one of the most common causes (6%) in European-derived populations.[7,8] Therefore, even partial prevention of myopia progression can provide important protection from pathological outcomes.[3]

How to increase students' outdoor time effectively and easily?

Time outdoors has been found to be a protective factor during the onset and progression of myopia in children in many previous studies.[9-15] In China, however, parents attach great importance to education, so the competition for education is very fierce and students' academic burden is heavy. This leads to poor compliance with increased outdoor time for students, especially during school time.

Outdoor time outside of school and parents

Previous interventions are focusing on increase in time outdoors or light exposure at school, and no intervention has been evaluated on increase in time outdoors outside school, such as weekends and holidays. Outside school, students' time is managed by their parents and there is still plenty of room for manoeuvre. At present, most parents of primary school students are between 20 and 40 years old. These young parents are more receptive to new things than their own parents. They may take advice in new ways to increase their children's outdoor time.

Smart phone users in China and short message service (SMS) intervention

In China, young parents all use smart phones and are familiar with WeChat and electronic payments. They spend a lot of time looking at their mobile phones every day. Some text messages from hospitals and schools about their children are easy for them to read and take seriously. We know that short message service (SMS) are being used more and more in public health. SMS has been found to be effective to

help people quit smoking,[16] to improve adherence in treatment of patients with HIV infection,[17] asthma,[18] type 2 diabetes,[19] as well as childhood cataract.[20] Can we use text alerts to increase the amount of time students spend outdoors outside of school? We think the idea is technically feasible. The effect of this method has not been reported before. For outdoor time measurement, we tend to use more accurate illumination records rather than questionnaires. Light meter (HOBO, Pendant temp/light Part # UA-002-64) is a kind of device with light sensor that has been used to measure light condition around plants, and has been used in measuring light exposure of children in recent studies.[21]

3. HYPOTHESIS

4.1 Hypothesis 1： Sending text messages to parents to remind them to take their children outside may increase the amount of time they spend outdoors.

4.2 Hypothesis 2： In the long run, it may be possible to slow the progression of myopia or delay the elongation of axial length by sending parents text messages reminding them to take their children outside more.

4. STUDY OBJECTIVES

4.1 General objectives

Our overall objective is to determine whether text message reminder could be used as easy intervention to increase children's outdoor time and then prevent myopia progression in a long term.

4.2 Specific objectives

Objective 1: To investigate whether text message reminder administered to parents could increase time outdoors of school-aged children.

Objective 2: To investigate whether text message reminder could increase the exposure of sunlight measured by a portable light meter in children

Objective 2: To determine whether text message reminder administrated to parents could prevent myopia progression of children in a long term.

5. DESIGN AND METHODOLOGY

5.1 Study design

This will be a 1-year randomized controlled trial and the participants will be followed after cessation of the trial. The text message intervention (short message service, SMS) will be implemented in the intervention group over the period of 1 year. The trial will be conducted in the Anyang city, where we have established a school-based cohort study on myopia of school-aged children, the Anyang Childhood Eye Study.[22] According to previous study on prevention and control of myopia in children, we chose students in grade 2 of primary school as the research participants, because children in this grade are more likely to have myopia.

We will continue to follow these children at the end of the trial to see if the intervention has a prolonged effect. In other words, could 1-year SMS intervention among parents have a lasting effect on the prevention and control of myopia in children?

5.2 Study site

This study will be carried out in Anyang, Henan province, where we have established a cohort of children with myopia, namely the Anyang Childhood Eye Study (ACES). The Anyang city (**Figure 2**) is a prefecture-level city, located in central China, with its economic development at the national average level, small population mobility and good representativeness and compliance.

Since we have been engaged in the epidemiological investigation of myopia here for many years and cooperated with the local Anyang Eye Hospital for many years,[22] the staff who participated in this study were all systematically trained. The Anyang Eye Hospital is the best local eye hospital, which can provide manpower and equipment support for this study.

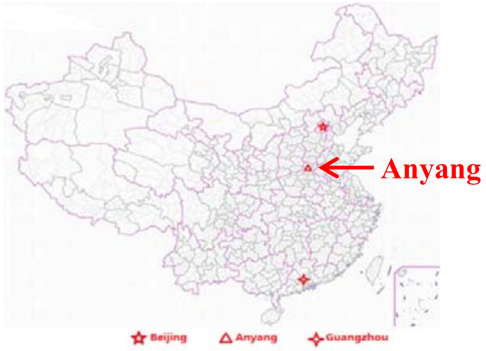

Figure 2. The study site of this study, Anyang city.

5.3 Study populations

According to the local statistical report, Anyang city had 528,965 primary school students in 2017. When we designed the cohort of Anyang Childhood Eye Study, we have randomly selected a total of 3,113 students from 11 primary schools.[22] In the implementation of this trial, we plan to further randomly select a certain number of grade 2 primary school students from the enrolled cohort (Figure 2).

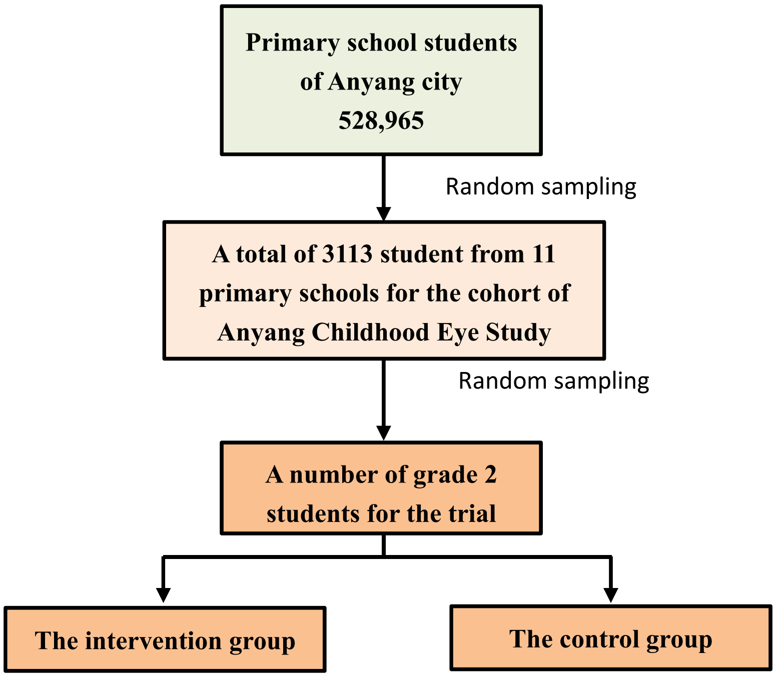

Figure 2. The study population and random sampling for this trial.

The reason for randomly selecting subjects in this study from the cohort of Anyang Childhood Eye Study was based on the following considerations. 1) the students enrolled in the cohort were randomly selected from more than 520,000 local students in Anyang, with good representativeness; 2) all the students in the cohort have undergone a cross-sectional survey and have the basis of previous data; 3) the preliminary investigation includes various ophthalmic examinations and questionnaires, covering all the examinations needed in this study. The compliance of the selected subjects will be better. 4) it is still relatively easy to follow up these subjects after one year of study.

5.4 Randomization strategy and sequence generation

Random allocation of the text message intervention will be done at the individual level. All children and parents were allocated by simple randomization (1:1) to the SMS intervention group or the control group. A randomization scheme will be computer-generated using SAS (v 9.3; SAS Institute, Inc., Cary, NC, USA) by a statistician.

5.5 Allocation concealment

The intervention assignment will be retained by the study statistician. The intervention assignment generated by the statistician is handed over to an independent staff member. The staff managed the parents' cellphone information independently and contacted the local telecom operator to send text messages regularly to parents assigned to the intervention group. This staff member does not participate in any other process of the study, such as checking, contacting schools, collecting data, etc., and does not communicate with other staff members.

5.6 Blinding

The children, their parents and study staff, including the investigators and outcome recorders, were masked to group allocation. However, the staff personnel in charge

of randomization (statistician) and sending SMS (an independent staff) were not masked.

5.7 Arms and Interventions

1) Intervention group: The parents of the intervention group will be administered with text message twice every day, which remind them to take their children outside for more sunshine and activities. One text message reminder is administrated to the parents at the noon when the children go home for lunch and have a rest for 2 hours. Another text message reminder is administrated to the parents after the school time when the children have some time available to play outside.

   The text message was as the following:

   Dear parents,

   After investigating your child's study and living environment, and comparing similar results in other areas or countries, we found that, in terms of your child's current eye condition, his/her outdoor activity time and ambient light intensity are relatively insufficient, which is likely to cause myopia onset or development. Therefore, from now on, please urge your children to get outdoors as much as possible and reduce the amount of time they spend on computer and TV.

   Thank you!

2) Control group: The parents in this group didn’t receive any text messages.

5.8 Study outcomes

Primary outcomes

The primary outcomes of this study will be changes in axial length and cycloplegic autorefraction. An ocular biometry system (Lenstar LS900; Haag-Streit, Koeniz,

Switzerland) will be used to measure axial length. Cycloplegic autorefraction will be performed after using cycloplegic eyedrops of 1% cyclopentolate.

Secondary outcomes

The second outcomes of this study will be outdoor light exposure and time outdoors of children. A light meter (HOBO, Pendant temp/light Part # UA-002-64) will be used to measure the light intensity (lux) around the child every 10 seconds during the 24 hours. Recording time was from 7 am to 7 pm on 3 randomly separated days, including 2 days during weekdays and 1 day at weekends, before and after the intervention.

5.9 Sample size

In our cohort study, we found a difference in axial elongation of 0.04 mm/year between the children with higher versus lower tertile of time outdoors. We take 0.04mm /year as a reference. In this study, it is designed to have a power of 90% and $\alpha = 0.05$ to detect the difference in treatment effect. Assuming an estimated loss to follow up of 20%, this study should enroll at least 210 children for the two groups.

In the local primary school in Anyang, there are about 50 students in each class. In order to ensure sufficient sample size, we plan to select five classes of primary school students as the research participants (about 250 students).

6. SELECTION AND ENROLLMENT OF SUBJECTS

Eligibility of the participants

Ages eligible for study: 6 years to 12 years

Genders eligible for study: both

7.1 Inclusion criteria:

1) Visual acuity: 20/20 or better in each eye;
2) Spherical error ranging from +1.5 D to -6.0 D and astigmatism less than 1.5 D in each eye, anisometropia less than 1.0 D between the two eyes;
3) No strabismus, amblyopia and any other ocular or systematic diseases that may affect the refractive development;

7.2 Exclusion criteria:

1) Currently using other interventions to control myopia progression (acupuncture, massage, drugs ear needles and so on)
2) Unable to cooperate with the ocular examination and questionnaire survey

7. STATISTICAL CONSIDERATIONS

8.1 Data Collection

Beside the light intensity data, all the other examination data will be collected using standard study forms and be independently entered into the database using Epidata software 3.1 (The Epidata Association, Odense, Denmark) by two designated clinic staff members who will have undergone training on how to use the study databases as well as on protection of human subjects. The data on light intensity will be collected by the light sensor (HOBO, Pendant temp/light Part # UA-002-64) and be transferred into excel files.

8.2 Data Storage and Security

All study computers and records will be stored in a secure room with access limited only to authorized staff. All computers and databases will be password-protected with limited access.

8.3 Data Analysis

Data analysis will be performed using SAS software on an intention-to-treat basis (significance level $p < 0.05$). Mean differences before and immediately after the interventions will be calculated. Baseline characteristics will be shown as mean ± standard deviation (SD) for continuous data including age, spherical equivalent, axial length, light exposure, time outdoors and near work load. As for participants' gender, the proportion of participants with myopia and number of myopic parents, n (%) of male and female and n (%) of participants in each group will be shown as baseline characteristics. Only the results of the right eye were used in the statistical analysis. If the values were normally distributed, analysis of Covariance (ANCOVA) was used otherwise nonparametric test would be used for analysis. Chi-square test was used to compare categorical outcomes.

9 HUMAN SUBJECTS

9.1 Institutional Review Board Review and Informed Consent

This protocol and the informed consent document and any subsequent modifications will be reviewed and approved by the Institutional Review Board (IRB) or ethics committee responsible for oversight of the study. A signed consent form will be obtained from the children's parents (or legal guardian, or person with power of attorney for children who cannot consent for themselves). The consent form will describe the purpose of this study, the procedures to be followed, and the risks and benefits of participating this study. A copy of the consent form will be given to the child, parent, or legal guardian, and this fact will be documented in the children's record.

9.2 Subject Confidentiality

All examinations, evaluation forms, reports, and other records that leave the site will be identified by coded number only to maintain subject confidentiality. All records will be kept locked. All computer entry and networking programs will be done with coded numbers only. Clinical information will not be released without written permission of the children's parents (or legal guardian), except as necessary for monitoring by the IRB.

9.3 Study Discontinuation

The study may be discontinued at any time by the IRB.

10 TIME FRAME

This trial will take 12 months to complete. All participants will be measured at baseline and 12 months. If conditions permit, the participant will be followed up for additional three years to observe the long-term effect. Table 1 summarizes measures and collection time points.

Table 1. Measurement schedule

MESASURES	BASELINE	12-MONTHS	ADDITIONAL FOLLOW-UP
PRIMARY OUTCOMES			
Light exposure	X	X	
Axial elongation	X	X	X
SECONDARY OUTCOMES			
Time outdoors	X	X	
Myopic shift	X	X	X
Parental myopia	X		

11 REFERENCES

References

1. Pan CW, Ramamurthy D, Saw SM. Worldwide prevalence and risk factors for myopia. Ophthalmic Physiol Opt 2012;32:3-16.
2. Vitale S, Ellwein L, Cotch MF, Ferris FL, 3rd, Sperduto R. Prevalence of refractive error in the United States, 1999-2004. Arch Ophthalmol 2008;126:1111-9.
3. Morgan IG, Ohno-Matsui K, Saw SM. Myopia. Lancet 2012;379:1739-48.
4. Liang YB, Friedman DS, Wong TY, et al. Prevalence and causes of low vision and blindness in a rural chinese adult population: the Handan Eye Study. Ophthalmology 2008;115:1965-72.
5. Xu L, Wang Y, Li Y, Cui T, Li J, Jonas JB. Causes of blindness and visual impairment in urban and rural areas in Beijing: the Beijing Eye Study. Ophthalmology 2006;113:1134 e1-11.
6. Hsu WM, Cheng CY, Liu JH, Tsai SY, Chou P. Prevalence and causes of visual impairment in an elderly Chinese population in Taiwan: the Shihpai Eye Study. Ophthalmology 2004;111:62-9.
7. Klaver CC, Wolfs RC, Vingerling JR, Hofman A, de Jong PT. Age-specific prevalence and causes of blindness and visual impairment in an older population: the Rotterdam Study. Arch Ophthalmol 1998;116:653-8.
8. Buch H, Vinding T, La Cour M, Appleyard M, Jensen GB, Nielsen NV. Prevalence and causes of visual impairment and blindness among 9980 Scandinavian adults: the Copenhagen City Eye Study. Ophthalmology 2004;111:53-61.
9. Rose KA, Morgan IG, Ip J, et al. Outdoor activity reduces the prevalence of myopia in children. Ophthalmology 2008;115:1279-85.
10. Sherwin JC, Reacher MH, Keogh RH, Khawaja AP, Mackey DA, Foster PJ. The Association between Time Spent Outdoors and Myopia in Children and Adolescents: A Systematic Review and Meta-analysis. Ophthalmology 2012;119:2141-51.
11. Wu PC, Tsai CL, Wu HL, Yang YH, Kuo HK. Outdoor Activity during Class Recess Reduces Myopia Onset and Progression in School Children. Ophthalmology 2013;120:1080-5.
12. Jones-Jordan LA, Sinnott LT, Cotter SA, et al. Time Outdoors, Visual Activity, and Myopia Progression in Juvenile-Onset Myopes. Invest Ophthalmol Vis Sci 2012;53:7169-75.
13. Guggenheim JA, Northstone K, McMahon G, et al. Time outdoors and physical activity as predictors of incident myopia in childhood: a prospective cohort study. Invest Ophthalmol Vis Sci 2012;53:2856-65.
14. Guo Y, Liu LJ, Xu L, et al. Outdoor Activity and Myopia among Primary Students in Rural and Urban Regions of Beijing. Ophthalmology 2012;120:277-83.
15. Dirani M, Tong L, Gazzard G, et al. Outdoor activity and myopia in Singapore teenage children. Br J Ophthalmol 2009;93:997-1000.
16. Rodgers A, Corbett T, Bramley D, et al. Do u smoke after txt? Results of a randomised trial of smoking cessation using mobile phone text messaging. Tobacco control 2005;14:255-61.
17. Lester RT, Ritvo P, Mills EJ, et al. Effects of a mobile phone short message service on antiretroviral treatment adherence in Kenya (WelTel Kenya1): a randomised trial. Lancet 2010;376:1838-45.
18. Strandbygaard U, Thomsen SF, Backer V. A daily SMS reminder increases adherence to asthma treatment: a three-month follow-up study. Respiratory medicine 2010;104:166-71.
19. Yoon KH, Kim HS. A short message service by cellular phone in type 2 diabetic patients for 12

months. Diabetes Res Clin Pract 2008;79:256-61.

20. Lin H, Chen W, Luo L, et al. Effectiveness of a short message reminder in increasing compliance with pediatric cataract treatment: a randomized trial. Ophthalmology 2012;119:2463-70.

21. Dharani R, Lee CF, Theng ZX, et al. Comparison of measurements of time outdoors and light levels as risk factors for myopia in young Singapore children. Eye (Lond) 2012;26:911-8.

22. Li SM, Liu LR, Li SY, et al. Design, methodology and baseline data of a school-based cohort study in central China: the Anyang Childhood Eye Study. Ophthalmic Epidemiol 2013;20:348-59.
