# Supplementary material for: Effect of Text Messaging Parents of School-Aged Children on Outdoor Time to Control Myopia: A Randomized Clinical Trial
Source: JAMA Pediatr. 2022 Sep 26;176(11):1077–83. doi: 10.1001/jamapediatrics.2022.3542 (PMC9513710; doi:10.1001/jamapediatrics.2022.3542)
Supplement: Supplement 2. — eMethods 1. Mixed-Effects Models eMethods 2. Structural Equation Model eMethods 3. R Script for Performing the Statistical Analysis eTable 1. Light Exposure and Time Outdoors Between the 2 Groups Following the Intervention eTable 2. Axial Elongation and Myopic Shift Among Subgroups of Nonmyopes, Premyopes, and Myopes During the 1-Year Intervention eFigure 1. Axial Elongation (A) and Myopic Shift (B) Following the 1-Year Intervention and for the Next 3 Years eFigure 2. Axial Elongation (A) and Myopic Shift (B) Among Subgroups of Nonmyopes, Premyopes, and Myopes Following the 1-Year Intervention and at the Next 3 Years [file jamapediatr-e223542-s002.pdf]

Supplemental Online Content

Li SM, Ran AR, Kang MT, et al; Anyang Childhood Eye Study Group. Effect of text messaging parents of school-aged children on outdoor time to control myopia: a randomized clinical trial. *JAMA Pediatr*. Published online September 26, 2022. doi:10.1001/jamapediatrics.2022.3542

eMethods 1. Mixed-Effects Models
eMethods 2. Structural Equation Model
eMethods 3. R Script for Performing the Statistical Analysis
eTable 1. Light Exposure and Time Outdoors Between the 2 Groups Following the Intervention
eTable 2. Axial Elongation and Myopic Shift Among Subgroups of Nonmyopes, Premyopes, and Myopes During the 1-Year Intervention
eFigure 1. Axial Elongation (A) and Myopic Shift (B) Following the 1-Year Intervention and for the Next 3 Years
eFigure 2. Axial Elongation (A) and Myopic Shift (B) Among Subgroups of Nonmyopes, Premyopes, and Myopes Following the 1-Year Intervention and at the Next 3 Years

This supplementary material has been provided by the authors to give readers additional information about their work.

© 2022 Li SM et al. *JAMA Pediatrics*.

eMethods 1. Mixed-Effects Models

The following regression formula was used for longitudinal data, with independent variables including time (years), SMS, primary outcomes at baseline (axial length, spherical equivalent refraction), light exposure (lux) and time outdoors (hours).

$$y_{it} = \beta_0 + \tau_{0i} + (\beta_1 + \tau_{1i}) \times time_{it} + (\beta_2 + \tau_{2i}) \times SMS_i + \beta_3 \times Baseline_i + \beta_4 \times light_i + \beta_5 \times outdoor_i + \varepsilon_{it}$$

1. Results of AL (axial length) as dependent variable.

As a result, SMS had a significant effect on AL between the two groups (P=0.0119). Baseline AL and time also had effects on AL (P=0.0000, P=0.0000).

```
Fixed effects:  y ~ time + SMS + AL_baseline + light + outdoor
                  Value Std.Error  DF  t-value p-value
(Intercept) -1.0816922 0.5282054 776 -2.04786  0.0409
time         0.3475820 0.0119928 776 28.98248  0.0000
SMS          0.0936679 0.0369816 254  2.53283  0.0119
AL_baseline  1.0431002 0.0228224 254 45.70515  0.0000
light        0.0003882 0.0007431 254  0.52249  0.6018
outdoor     -0.0211538 0.1386697 254 -0.15255  0.8789
```

2. Results of RA (myopic shift) as dependent variable.

As a result, SMS had no significant effect on RA between the two groups (P=0.2298). Baseline RA and time had effects on RA (P=0.0000, P=0.0001).

```
Fixed effects:  y ~ time + SMS + RA_baseline + light + outdoor
                 Value Std.Error  DF   t-value p-value
(Intercept) -0.771203 0.9576167 776 -0.805336  0.4209
time        -0.646898 0.1607561 776 -4.024100  0.0001
SMS          0.761054 0.6321692 254  1.203877  0.2298
RA_baseline  1.176929 0.2645696 254  4.448468  0.0000
light       -0.011928 0.0137284 254 -0.868847  0.3858
outdoor      3.691433 2.5995615 254  1.420021  0.1568
```

© 2022 Li SM et al. *JAMA Pediatrics*.

eMethods 2. Structural Equation Model

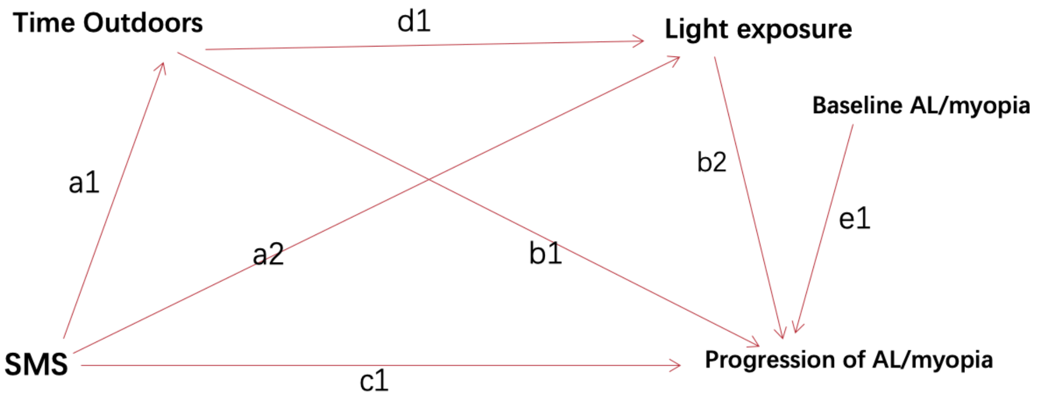

Considering that there is both a direct effect of SMS on AL/myopia progression and an indirect effect by affecting light exposure or outdoor time, which in turn affects AL/myopia progression, we designed a mediating effect pathway analysis based on structural equation modeling (SEM) as shown in above Figure. Each edge of the graph represents a direct effect, and some indirect effects are defined as follows.

Ind1=a1*b1: Indirect effect of SMS on AL/myopia progression through outdoor time.

Ind2=a2*b2: Indirect effect of SMS on AL/myopia progression through light exposure.

secondInd1=a1*d1*b2: Indirect effects of SMS on AL/myopia progression via outdoor time-light exposure.

c=c1+a1*b1+a2*b2+a1*d1*b2: Total effect of SMS on AL/myopia progression.

3. Results of AL (axial length) as dependent variable.

As a result, SMS had a significant direct effect on AL between the two groups (P=0.035).There were no other significant direct or indirect effects.

© 2022 Li SM et al. *JAMA Pediatrics*.

```
Regressions:
                   Estimate  Std.Err  z-value  P(>|z|) ci.lower ci.upper
  y.AL ~
    outdoor   (b1)   -0.421    0.255   -1.652    0.099   -0.920    0.078
    light     (b2)    0.002    0.001    1.494    0.135   -0.001    0.005
    SMS       (c1)    0.127    0.062    2.065    0.039    0.006    0.248
    AL_baseln (e1)    0.196    0.040    4.874    0.000    0.117    0.275
  outdoor ~
    SMS       (a1)   -0.012    0.038   -0.331    0.741   -0.086    0.061
  light ~
    SMS       (a2)    1.325    2.837    0.467    0.641   -4.235    6.885
    outdoor   (d1)  173.727    4.683   37.096    0.000  164.548  182.906

Variances:
                   Estimate  Std.Err  z-value  P(>|z|) ci.lower ci.upper
   .y.AL              0.244    0.021   11.380    0.000    0.202    0.286
   .outdoor           0.092    0.008   11.380    0.000    0.076    0.107
   .light           520.809   45.766   11.380    0.000  431.109  610.509

Defined Parameters:
                   Estimate  Std.Err  z-value  P(>|z|) ci.lower ci.upper
    Ind1              0.005    0.016    0.325    0.745   -0.026    0.037
    Ind2              0.003    0.006    0.446    0.656   -0.009    0.014
    secondInd1       -0.004    0.013   -0.323    0.747   -0.031    0.022
    c                 0.131    0.062    2.113    0.035    0.009    0.252
```

4. Results of RA (spherical equivalent refraction) as dependent variable.

As a result, SMS had no significant direct effect on RA between the two groups (P=0.427).There were no other significant direct or indirect effects.

```
Regressions:
                   Estimate  Std.Err  z-value  P(>|z|) ci.lower ci.upper
  y.RA ~
    outdoor   (b1)    7.303    2.896    2.521    0.012    1.626   12.980
    light     (b2)   -0.030    0.015   -1.960    0.050   -0.060   -0.000
    SMS       (c1)    0.632    0.704    0.898    0.369   -0.748    2.013
    RA_baseln (e1)    0.296    0.295    1.005    0.315   -0.282    0.874
  outdoor ~
    SMS       (a1)   -0.012    0.038   -0.331    0.741   -0.086    0.061
  light ~
    SMS       (a2)    1.325    2.837    0.467    0.641   -4.235    6.885
    outdoor   (d1)  173.727    4.683   37.096    0.000  164.548  182.906

Variances:
                   Estimate  Std.Err  z-value  P(>|z|) ci.lower ci.upper
   .y.RA             31.556    2.773   11.380    0.000   26.121   36.991
   .outdoor           0.092    0.008   11.380    0.000    0.076    0.107
   .light           520.809   45.766   11.380    0.000  431.109  610.509

Defined Parameters:
                   Estimate  Std.Err  z-value  P(>|z|) ci.lower ci.upper
    Ind1             -0.091    0.277   -0.328    0.743   -0.634    0.452
    Ind2             -0.040    0.087   -0.454    0.650   -0.211    0.132
    secondInd1        0.065    0.199    0.326    0.744   -0.325    0.454
    c                 0.567    0.713    0.794    0.427   -0.832    1.965
```

© 2022 Li SM et al. *JAMA Pediatrics*.

eMethods 3. R Script for Performing the Statistical Analysis

```
library(tidyverse)
library(nlme)
library(lavaan)
data <- read.csv("data.csv")
light <- read.csv("light.csv")


light$WEINO = light$WEINO-1
NNAA = data[2,15]
aa = as.data.frame(matrix(NNAA, nrow = dim(data)[1], ncol = dim(light)[2]))
names(aa) = names(light)
aa[match(light$WEINO,data$WEINO),] = light
data.all = cbind(data, aa)
data0 = data.all[,c(1:20,22:27)]
data0$GROUP = data0$GROUP - 1
names(data0)[match("GROUP",names(data0))] = "SMS"
names(data0)[match("af_weekly",names(data0))] = "outdoor"
names(data0)[match("light_af_weekly_adj",names(data0))] = "light"
names(data0)[match("AL_2",names(data0))] = "AL_baseline"
names(data0)[match("RA_2",names(data0))] = "RA_baseline"


################ MLM ######################
### AL
nas <- c(names(data0)[c(1,1+2*c(2:6))],"SMS","outdoor","light")
data.AL = data0[,nas]
data.ana = data.AL
names(data.ana)
na.n = apply(data.ana, 1, function(x) sum(is.na(x)))
data.ana = data.ana[na.n==0,]
data.ana.long <- data.ana %>% gather(key = "y.names", value = "y", -WEINO,-
AL_baseline,-SMS,-outdoor,-light)
time = sort(rep(1:4,dim(data.ana)[1]))
data.ana.long <- cbind(data.ana.long,time)
names(data.ana.long)


lme01 <- lme(
  y ~ time + SMS + AL_baseline + light + outdoor,
  random = ~ 1 + time + SMS | WEINO,
  data = data.ana.long)
```

© 2022 Li SM et al. *JAMA Pediatrics*.

```
summary(lme01)

lme02 <- lme(
   y ~ time + SMS + AL_baseline,
   random = ~ 1 + time + SMS | WEINO,
   data = data.ana.long)
summary(lme02)

### RA
nas <- c(names(data0)[c(1,0+2*c(2:6))],"SMS","outdoor","light")
data.RA = data0[,nas]
data.ana = data.RA
names(data.ana)
na.n = apply(data.ana, 1, function(x) sum(is.na(x)))
data.ana = data.ana[na.n==0,]

data.ana.long <- data.ana %>% gather(key = "y.names", value = "y", -WEINO,-RA_baseline,-SMS,-outdoor,-light)
t = sort(rep(1:4,dim(data.ana)[1]))
data.ana.long <- cbind(data.ana.long,t)
names(data.ana.long)


lme3 <- lme(
   y ~ time + SMS + RA_baseline + light + outdoor,
   random = ~ 1 + time | WEINO,
   data = data.ana.long)
summary(lme3)

lme4 <- lme(
   y ~ time + SMS + RA_baseline,
   random = ~ 1 + time | WEINO,
   data = data.ana.long)
summary(lme4)


################ SEM #######################
y.RA = data0[,"RA_6"] - data0[,"RA_baseline"]
y.AL = data0[,"AL_6"] - data0[,"AL_baseline"]
nas <- c("RA_baseline","AL_baseline","RA_3","AL_3","SMS","outdoor","light","WEINO")
data0.sem = cbind(y.RA,y.AL,data0[,nas])
na.n = apply(data0.sem, 1, function(x) sum(is.na(x)))
data0.sem = data0.sem[na.n==0,]
```

© 2022 Li SM et al. *JAMA Pediatrics*.

```
## AL
multipleMediation <- ' y.AL~b1*outdoor+b2*light+c1*SMS + e1*AL_baseline
outdoor~a1*SMS
light~a2*SMS+d1*outdoor
Ind1:=a1*b1
Ind2:=a2*b2
secondInd1:=a1*d1*b2
c:=c1+a1*b1+a2*b2+a1*d1*b2 '
fit.AL <- sem( model=multipleMediation, data= data0.sem)
summary(fit.AL,ci=T)


## RA
multipleMediation <- ' y.RA~b1*outdoor+b2*light+c1*SMS + e1*RA_baseline
outdoor~a1*SMS
light~a2*SMS+d1*outdoor
Ind1:=a1*b1
Ind2:=a2*b2
secondInd1:=a1*d1*b2
c:=c1+a1*b1+a2*b2+a1*d1*b2 '
fit.RA <- sem( model=multipleMediation, data= data0.sem)
summary(fit.RA,ci=T)
```

© 2022 Li SM et al. *JAMA Pediatrics*.

eTable 1. Light Exposure and Time Outdoors Between the 2 Groups Following the Intervention

	**SMS group Mean (SD)**	**Control group Mean (SD)**	**Difference† (95% CI)**
Light exposure *(Lux)*			
Weekend	46 (66)	28 (48)	17.73 (3.26~32.21)
Weekdays	173 (70)	179 (76)	–5.77 (–23.62~12.09)
Weekly	953 (409)	946 (401)	7.80 (–90.89~106.51)
Difference* (95% CI)	350 (280~421)	320 (240~399)	
Time outdoors (hours)			
Weekend	0.34 (0.58)	0.18 (0.36)	0.16 (0.02~0.29)
Weekday	0.85 (0.35)	0.89 (0.37)	–0.03(–0.12~0.06)
Weekly	4.80 (2.27)	4.71 (1.99)	0.10 (–0.43~0.62)
Difference* (95% CI)	1.26 (0.87~1.66)	1.06 (0.66~1.46)	

*Difference in weekly values of each group before the intervention subtracted from those after the intervention. †Difference between the two groups. SMS: short message service. SD: standard deviation.

© 2022 Li SM et al. *JAMA Pediatrics*.

eTable 2. Axial Elongation and Myopic Shift Among Subgroups of Nonmyopes, Premyopes, and Myopes During the 1-Year Intervention

	**SMS group (n=133)**	**Control group (n=133)**	**All (n=266)**
Axial elongation, mean (95% CI), mm			
Non-myopes	0.21 (0.20 to 0.23)	0.25 (0.23 to 0.28)	0.23 (0.22 to 0.25)
Pre-myopes	0.52 (0.41 to 0.63)	0.40 (0.34 to 0.47)	0.46 (0.39 to 0.52)
Myopes	0.41 (0.35 to 0.47)	0.49 (0.42 to 0.56)	0.46 (0.41 to 0.51)
Myopic shift, mean (95% CI), D			
Non-myopes	-0.26 (-0.32 to -0.20)	-0.23 (-0.38 to -0.08)	-0.25 (-0.33 to -0.17)
Pre-myopes	-0.94 (-1.14 to -0.74)	-0.96 (-1.15 to -0.77)	–0.99 (-1.14 to -0.84)
Myopes	-0.78 (-0.97 to -0.58)	-1.04 (-1.21 to -0.87)	–0.99 (-1.13 to -0.84)

SMS: short message service.

© 2022 Li SM et al. *JAMA Pediatrics*.

eFigure 1. Axial Elongation (A) and Myopic Shift (B) Following the 1-Year Intervention and for the Next 3 Years

'*' indicates statistical significance between the two groups. Error bars indicate standard deviations. SMS = short message service/text message. Years 1-3 refer to the years of follow-up.

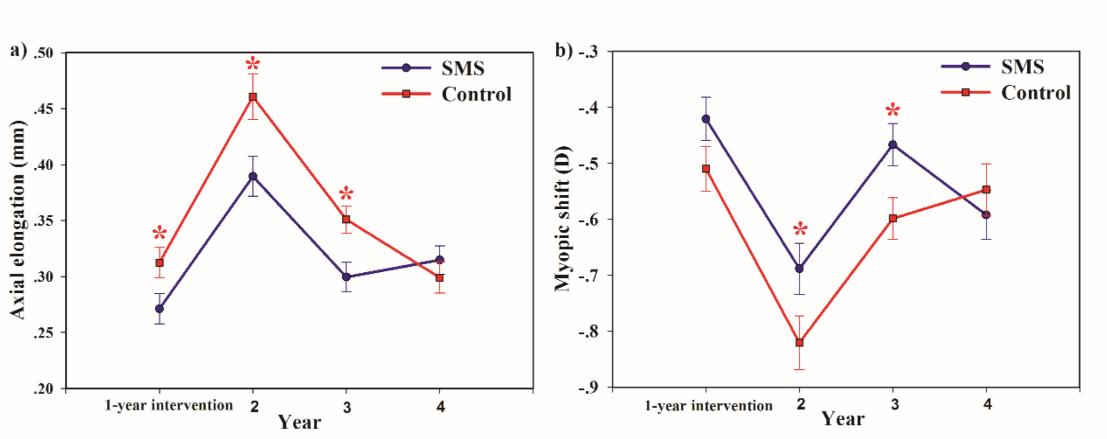

© 2022 Li SM et al. *JAMA Pediatrics*.

eFigure 2. Axial Elongation (A) and Myopic Shift (B) Among Subgroups of Nonmyopes, Premyopes, and Myopes Following the 1-Year Intervention and at the Next 3 Years

Non-myopes (dashed lines) had lower axial elongation and myopic shift than pre-myopes (dotted lines) and myopes (solid lines) over the course of the study. Years 1-3 refer to the years of follow-up. Error bars indicate standard deviations.

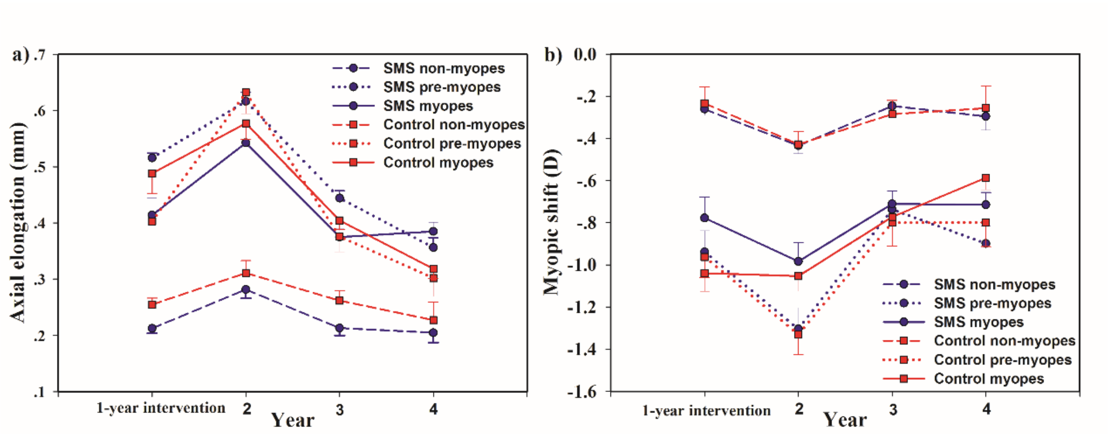

© 2022 Li SM et al. *JAMA Pediatrics*.
