# Supplementary material for: Effect of Text Messaging Parents of School-Aged Children on Outdoor Time to Control Myopia: A Randomized Clinical Trial
Source: JAMA Pediatr. 2022 Sep 26;176(11):1077–83. doi: 10.1001/jamapediatrics.2022.3542 (PMC9513710; doi:10.1001/jamapediatrics.2022.3542)
Supplement: Supplement 3. — The Anyang Childhood Eye Study Group [file jamapediatr-e223542-s003.pdf]

Supplemental Online Content: Nonauthor Collaborators

*Indicates required information. Only first name, last name, and suffix will appear in PubMed.

***Group Name(s): The Anyang Childhood Eye Study Group**

| ***First Name and Middle Initial(s)** | ***Last Name** | ***Suffix (eg, Jr, III)** | Academic Degrees | Institution | Location (city, state/province, country) | Role or Contribution, eg, chair, principal investigator | Group (if more than 1 Group listed in the byline) and/or Subgroup (eg, Steering Committee) |
|---|---|---|---|---|---|---|---|
| Paul | Mitchell | | MD, PhD | Department of Ophthalmology, Centre for Vision Research, Westmead Millennium Institute, University of Sydney | Sydney, New South Wales, Australia | Co-Principal investigator, designed the Anyang Childhood Eye Study | |
| Yazhou | Ji | | MD | Anyang Eye Hospital | Anyang city, Henan province, China | Local coordinator | |
| Weixin | He | | | Anyang Health Bureau (Deputy director) | Anyang city, Henan province, China | Local coordinator | |
| Hailin | Meng | | MD | Anyang Eye Hospital | Anyang city, Henan province, China | Local coordinator | |
| Yongfang | Tu | | MD | Anyang Eye Hospital | Anyang city, Henan province, China | Local coordinator | |
| Hongyuan | Wang | | MM | Department of Epidemiology and Health Statistics, School of Public Health, Peking University | Beijing, China | Statistician, Sampling | |
| Jing | Fu | | MD, PhD | Beijing Tongren Eye Center, Beijing Tongren Hospital, Capital Medical University | Beijing, China | Study staff | |
| Siyuan | Li | | MD, PhD | Department of Ophthalmology, Beijing Children Hospital, Capital Medical University | Beijing, China | Study staff | |
| Zhou | Yang | | MD, PhD | Department of Ophthalmology, Children's Hospital Attached to The Capital Institute of Pediatrics | Beijing, China | Study staff | |
| Jiyuan | Guo | | MD | Anyang Eye Hospital | Anyang city, Henan province, China | Study staff | |
| Shiqiang | Zhao | | MM | Beijing Tongren Eye Center, Beijing Tongren Hospital, Capital Medical University | Beijing, China | Study staff | |

Supplemental Online Content: Nonauthor Collaborators

*Indicates required information. Only first name, last name, and suffix will appear in PubMed.

| ***First Name and Middle Initial(s)** | ***Last Name** | ***Suffix (eg, Jr, III)** | Academic Degrees | Institution | Location (city, state/province, country) | Role or Contribution, eg, chair, principal investigator | Group (if more than 1 Group listed in the byline) and/or Subgroup (eg, Steering Committee) |
|---|---|---|---|---|---|---|---|
| Bidan | Zhu | | MD | Beijing Tongren Eye Center, Beijing Tongren Hospital, Capital Medical University | Beijing, China | Study staff | |
| Yunyun | Sun | | MD, PhD | Beijing Tongren Eye Center, Beijing Tongren Hospital, Capital Medical University | Beijing, China | Study staff | |
| Bo | Meng | | MD, PhD | Beijing Tongren Eye Center, Beijing Tongren Hospital, Capital Medical University | Beijing, China | Study staff | |
| Yizao | Zhang | | MD | Anyang Eye Hospital | Anyang city, Henan province, China | Study staff | |
| Jinling | Li | | MD | Anyang Eye Hospital | Anyang city, Henan province, China | Study staff | |
