# Supplementary material for: Effect of Text Messaging Parents of School-Aged Children on Outdoor Time to Control Myopia: A Randomized Clinical Trial
Source: JAMA Pediatr. 2022 Sep 26;176(11):1077–83. doi: 10.1001/jamapediatrics.2022.3542 (PMC9513710; doi:10.1001/jamapediatrics.2022.3542)
Supplement: Supplement 4. — Data Sharing Statement [file jamapediatr-e223542-s004.pdf]

Data Sharing Statement

Li. Effect of Text Messaging Parents of School-Aged Children on Outdoor Time to Control Myopia. *JAMA Pediatr*. Published September 26, 2022. doi:10.1001/jamapediatrics.2022.3542

Data
Data available: Yes
Data types: Deidentified participant data, Data dictionary
How to access data: lishiming81@163.com
When available: With publication

Supporting Documents
Document types: Statistical/analytic code
How to access documents: lishiming81@163.com
When available: With publication

Additional Information
Who can access the data: Shi-Ming Li, Ningli Wang
Types of analyses: Conduct joint analysis of data
Mechanisms of data availability: with a signed data access agreement
